# Tim-3 Negatively Regulates IL-12 Expression by Monocytes in HCV Infection

**DOI:** 10.1371/journal.pone.0019664

**Published:** 2011-05-26

**Authors:** Ying Zhang, Cheng J. Ma, Jia M. Wang, Xiao J. Ji, Xiao Y. Wu, Zhan S. Jia, Jonathan P. Moorman, Zhi Q. Yao

**Affiliations:** 1 Medical Service, Department of Veterans Affairs, James H. Quillen VA Medical Center, Johnson City, Tennessee, United States of America; 2 Division of Infectious Diseases, Department of Internal Medicine, James H. Quillen College of Medicine, East Tennessee State University, Johnson City, Tennessee, United States of America; 3 Department of Infectious Diseases, Tangdu Hospital, The Fourth Military Medical University, Xi'an, China; Centre de Recherche Public de la Santé (CRP-Santé), Luxembourg

## Abstract

T cell immunoglobulin and mucin domain-containing protein 3 (Tim-3) is a newly identified negative immunomodulator that is up-regulated on dysfunctional T cells during viral infections. The expression and function of Tim-3 on human innate immune responses during HCV infection, however, remains poorly characterized. In this study, we report that Tim-3 is constitutively expressed on human resting CD14^+^ monocyte/macrophages (M/M_Ø_) and functions as a cap to block IL-12, a key pro-inflammatory cytokine linking innate and adaptive immune responses. Tim-3 expression is significantly reduced and IL-12 expression increased upon stimulation with Toll-like receptor 4 (TLR4) ligand - lipopolysaccharide (LPS) and TLR7/8 ligand - R848. Notably, Tim-3 is over-expressed on un-stimulated as well as TLR-stimulated M/M_Ø_, which is inversely associated with the diminished IL-12 expression in chronically HCV-infected individuals when compared to healthy subjects. Up-regulation of Tim-3 and inhibition of IL-12 are also observed in M/M_Ø_ incubated with HCV-expressing hepatocytes, as well as in primary M/M_Ø_ or monocytic THP-1 cells incubated with HCV core protein, an effect that mimics the function of complement C1q and is reversible by blocking the HCV core/gC1qR interaction. Importantly, blockade of Tim-3 signaling significantly rescues HCV-mediated inhibition of IL-12, which is primarily expressed by Tim-3 negative M/M_Ø_. Tim-3 blockade reduces HCV core-mediated expression of the negative immunoregulators PD-1 and SOCS-1 and increases STAT-1 phosphorylation. Conversely, blocking PD-1 or silencing SOCS-1 gene expression also decreases Tim-3 expression and enhances IL-12 secretion and STAT-1 phosphorylation. These findings suggest that Tim-3 plays a crucial role in negative regulation of innate immune responses, through crosstalk with PD-1 and SOCS-1 and limiting STAT-1 phosphorylation, and may be a novel target for immunotherapy to HCV infection.

## Introduction

HCV is a serious and growing threat to public health, affecting approximately 4 million U.S. citizens and 200 million people worldwide [1]. The most remarkable feature of this blood-borne virus is its ability to evade host immunity, resulting in over 80% of infected individuals developing chronic infection that is associated with liver cirrhosis and hepatocellular carcinoma – thus becoming a leading cause for liver transplantation [1]. Unfortunately, the current standard treatment with pegylated interferon and ribavirin (IFN/RBV) has limited effectiveness (less than 50% respond) for the most prevalent viral genotypes (1a/1b) in the U.S [1]. No vaccine is currently available, in part due to our incomplete understanding of HCV-host interactions that lead to viral persistence.

Tim-3 is a type 1 membrane protein with a structurally conserved immunoglobulin variable (IgV) domain and mucin stalk that connects to an intracellular tail [2]. Tim-3 was initially identified expressed on activated Th1 cells, rather than Th2 cells, and the interaction between Tim-3 and its ligand, galectin-9 (gal-9), was shown to inhibit Th1 responses and induce cell death in individuals with autoimmune disorders [3]–[6]. Recently, Tim-3 has been found to be over-expressed on T cells in chronic viral infections, and its blockade rescued the exhausted virus- specific CD4^+^ and CD8^+^ T cell functions [7]–[10]; meanwhile, kupffer cell-derived galectin-9 (gal-9, Tim-3 ligand) has also been shown to play a role in regulation of T cell immunity in HCV infection [11]. Thus, the Tim-3/gal-9 pathway appears to function as negative signaling and play an important role in T cell dysfunction during chronic viral infections.

In addition to T cells, Tim-3 expression has recently been shown on innate immune cells, notably antigen presenting cells (APCs), and has more complex functions in immune dysregulation [12]–[15]. While extensive studies have shown Tim-3 as an inhibitory molecule on Th1/Tc1 cells, its role in M/M_Ø_ as well as maturation and function of dendritic cells (DC) is rather controversial. On the one hand, Tim-3 has been shown to negatively regulate macrophage activation, and Tim-3 signaling on cells of the innate immune system critically influences regulation of adaptive immune responses [12]–[13]. On the other hand, Tim-3 and gal-9 has been reported to induce maturation of human monocyte-derived DC (MDDC) and promote phagocytosis of apoptotic cells and cross-presentation of dying cell-associated antigen to T cells [14]–[16]. The expression and function of Tim-3, and its relationship with other negative immunoregulators such as programmed death-1 (PD-1) and suppressor of cytokine-1 (SOCS-1), in innate immune regulation during HCV infection remain unknown. In this report, we assessed the expression and effect of Tim-3 on human M/M_Ø_ and IL-12 regulation during chronic HCV infection. We found that Tim-3 is over-expressed on both un-stimulated and TLR-stimulated M/M_Ø_ and is negatively associated with the impaired IL-12 production in chronically HCV-infected individuals when compared to healthy subjects. HCV (core) increases Tim-3 expression and inhibits IL-12 production in primary M/M_Ø_ or monocytic THP-1 cells, an effect that mimics C1q and is reversible by blocking the HCV core/gC1qR interaction. Importantly, blockade of Tim-3 signaling significantly improves the HCV-mediated suppression of IL-12, which is primarily expressed by Tim-3 negative M/M_Ø_. We also found that Tim-3 alters the expressions of PD-1 and SOCS-1 to coordinately inhibit M/M_Ø_ IL-12 production by limiting the STAT-1 phosphorylation in M/M_Ø_. These findings suggest that Tim-3 is able to cooperative with other inhibitory molecules and plays a crucial role in negative regulation of innate immune responses during chronic viral infection.

## Results

### Tim-3 is over-expressed on resting and activated CD14^+^ M/M_Ø_ and is associated with diminished IL-12 expression in chronic HCV infection

**Figure 1:**
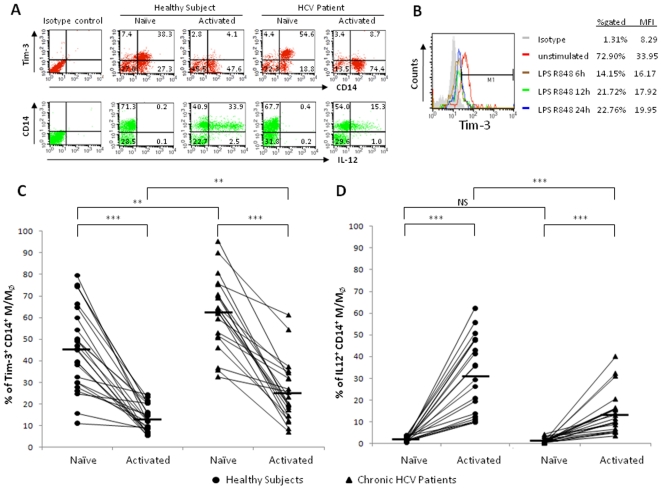
Tim-3 is over-expressed and IL-12 expression suppressed on CD14^+^ M/M_Ø_ in chronically HCV-infected individuals. PBMC from chronically HCV-infected subjects (n = 21) and healthy subjects (n = 12) were stimulated with or without TLR ligands LPS and R848 for 18 h, followed by immunostaining with antibodies against Tim-3, IL-12 and CD14. Tim-3 and IL-12 expressions in un-stimulated and LPS/R848-stimulated CD14^+^ M/M_Ø_ were examined by flow cytometry. A) Representative flow cytometric dot plots measuring Tim-3 expression and IL-12 production in CD14^+^ M/M_Ø_. B) Time-course of Tim-3 expression in primary healthy M/M_Ø_. PBMC stimulated with LPS and R848 for the indicated times. Tim-3 expression on CD14^+^ M/M_Ø_ was assessed by flow cytometry; corresponding changes in % gated and MFI are shown with isotype control. C) Summary data of the percentage of Tim-3^+^ and D) IL-12^+^ cells in CD14^+^ M/M_Ø_ of healthy subjects and chronically HCV-infected individuals, in naïve and TLR-activated state, are shown. Each symbol represents an individual subject, and the horizontal bars represent median values. The *p* value (**<0.01; ***<0.001) is denoted above the group of study subjects.

Tim-3 has been shown to be over-expressed on and involved in inhibiting viral specific T cell responses during HCV infection [9]. The expression and role of Tim-3 in regulation of M/M_Ø_ function during HCV infection remains undefined. To address this, Tim-3 expression, along with intracellular IL-12 production, in resting and TLR-stimulated M/M_Ø_ of chronically HCV-infected patients and healthy subjects, were examined by flow cytometry. As shown in the representative dot plots, time-course, and summary data of Tim-3 expression on CD14^+^ M/M_Ø_ of healthy subjects (n = 12, multiple assays, p<0.001) and HCV-infected individuals (n = 21, p<0.001) in Fig. 1A through D, Tim-3 is constitutively expressed on resting M/M_Ø_; its expression significantly decreases upon cell activation, as early as 6 h, after stimulation with Toll-like receptor 4 (TLR4) ligand - lipopolysaccharide (LPS) and TLR7/8 ligand - R848, which can synergistically activate primary M/M_Ø_ to produce IL-12 [17], [18]. Notably, chronically HCV-infected individual exhibits significantly elevated Tim-3 expression on CD14^+^ M/M_Ø_, in both the un-stimulated and TLR-stimulated states, when compared to healthy subjects. In contrast to the elevated Tim-3 expression, IL-12 expression is barely detectable in resting M/M_Ø_; its expression is significantly increased in CD14^+^ M/M_Ø_ following TLR stimulation, but to a lesser extent and leading to an impaired IL-12 expression in the setting of chronic HCV infection compared to healthy subjects.

**Figure 2:**
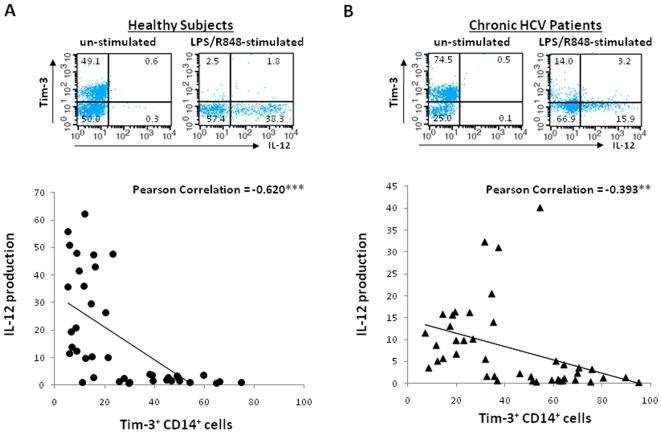
Tim-3 expression is inversely associated with IL-12 production in M/M_Ø_. Tim-3 expression and IL-12 production in CD14^+^ M/M_Ø_ were measured by flow cytometry triple immunostaining in healthy subjects (A) and chronically HCV-infected individuals (B). Representative dot plots to show the relationship of Tim-3 and IL-12 expressions in CD14^+^ M/M_Ø_ in un-stimulated and TLR-stimulated are shown above. The correlation between Tim-3 expression and IL-12 production in M/M_Ø_, evaluated by Pearson Correlation with 2-tailed significance (**<0.01, ***<0.001) denoted in the upper right corner of each analysis, is shown below.

To further determine whether Tim-3 expression correlates with IL-12 production in M/M_Ø_, we examined their expressions in all participants by flow cytometry at single cell basis with triple immunostaining and Pearson correlation analysis. As shown in the representative dot plot and summary data in Fig. 2A and B, Tim-3 is highly expressed on un-stimulated “naïve” CD14^+^ M/M_Ø_, in peripheral blood of both healthy subjects and chronically HCV-infected individuals, which produce negligible IL-12; once TLR-stimulated, Tim-3 expression is diminished, which is very significantly and inversely correlated to M/M_Ø_ IL-12 expression, primarily by CD14^+^Tim-3^−^ M/M_Ø_.

### Tim-3 and IL-12 expressions are differentially regulated in M/M_Ø_ co-cultured with hepatocytes expressing live HCV

**Figure 3:**
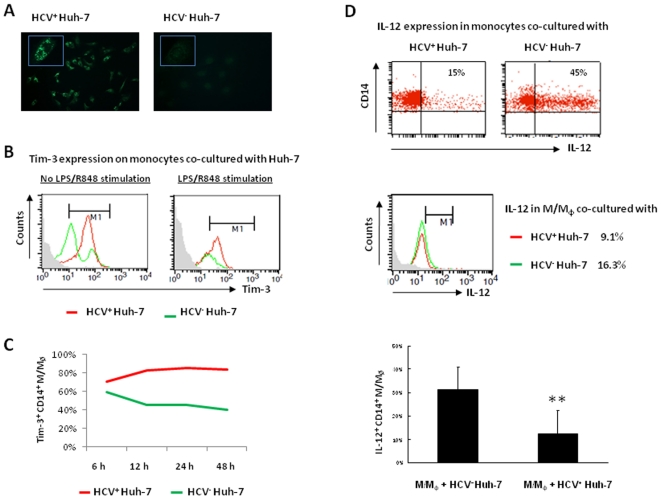
Tim-3 and IL-12 expression in M/M_Ø_ is differentially regulated by hepatocytes expressing HCV. A) Immunofluoresence staining of HCV core protein in Huh-7 cells 48 h after HCV-JFH-1 (left) or mock (right) transfection. Higher magnificent imaging (×40) is inserted at upper left corner. B) Tim-3 expression on M/M_Ø_ co-cultured with HCV-transfected (red line) or mock-transfected (green line) Huh-7 cells without (left) or with (right) LPS/R848 stimulation. C) Representative Time-course of Tim-3 expression on CD14^+^ M/M_Ø_ co-cultured with HCV^+^ Huh-7 versus HCV^−^ Huh-7 cells from two reproducible experiments. D) IL-12 expression in M/M_Ø_ co-cultured with HCV^+^ Huh-7 versus HCV^−^ Huh-7 cells, shown as dot plot, histogram, and bar figure in multiple experiments.

The observed Tim-3 up-regulation on M/M_Ø_ during chronic HCV infection might be a result rather than a cause of IL-12 suppression. To further elucidate the role of HCV in regulation of Tim-3 and IL-12 expression and to more accurately mimic the *in vivo* setting of chronic HCV infection, we employed a newly established cell culture system by transfecting Huh-7 hepatocytes with the HCV-JFH-1 strain *in vitro* [19], [20] As shown in Fig. 3A, HCV core as well as NS5 protein (Fig.S1A) is detected in HCV-JFH-1-transfected Huh-7 hepatocytes, but not in mock-transfected controls, by immunofluorescent staining. HCV core mRNA is also detected by RT-PCR in the supernatant of the HCV-transfected Huh-7 cells but not in the culture of controls (Fig.S1B). Additionally, uninfected Huh-7 cells can be infected by the supernatant of JFH-1-transfected Huh-7 cells (Fig.S1C), suggesting that live HCV particles are secreted from the HCV mRNA-transfected hepatocytes. We then incubated purified healthy M/M_Ø_ with Huh-7 cells 48 h after HCV transfection, followed by detection of Tim-3 and IL-12 expressions in M/M_Ø_ with or without TLR stimulation for 18 h. As shown in Fig. 3B, Tim-3 expression is found to be up-regulated on purified M/M_Ø_ co-cultured with hepatocytes expressing live HCV when compared with HCV^−^ hepatocytes, in both the un-stimulated and LPS/R848-stimulated state. As described above, Tim-3 is expressed at relatively high levels on M/M_Ø_ cultured with hepatocytes without TLR stimulation (Fig. 3B, left panel) when compared to those with LPS/R848 stimulation for 18 h (Fig. 3B, right panel); however, in either scenario, Tim-3 is up-regulated by HCV exposure. To determine the dynamics of Tim-3 up-regulation by HCV, we kinetically examined Tim-3 expression on M/M_Ø_ at various time-points after co-culture with HCV^+^ Huh-7 versus HCV^−^ Huh-7 without TLR stimulation. As shown in Fig. 3C, at all time-points examined, the expression of Tim-3 on CD14^+^ M/M_Ø_ incubated with HCV-expressing Huh-7 cells is remarkably higher than those co-cultured with HCV^−^ hepatocytes. We also examined IL-12 expression in M/M_Ø_ incubated with HCV^+^ hepatocytes or HCV^−^ hepatocytes for 18 h, as shown in a Fig. 3D dot plot, histogram, and bar figure derived from multiple repeated experiments, HCV^+^ Huh-7 significantly inhibits IL-12 production by CD14^+^ M/M_Ø_.

### HCV core protein up-regulates Tim-3 expression and down-regulates IL-12 production in primary M/M_Ø_ and THP-1 cells

**Figure 4:**
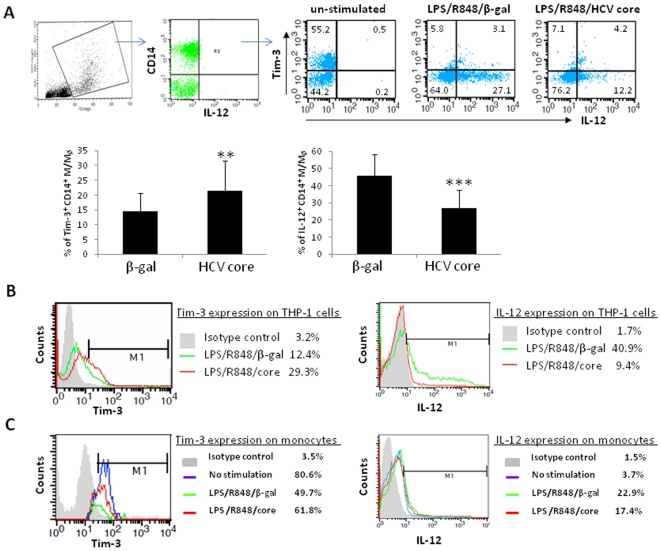
Tim-3 and IL-12 expression in primary CD14^+^ M/M_Ø_ and THP-1 cells are altered by HCV core treatment. A) Healthy PBMC were treated with β-gal or HCV core and LPS/R848 for 18 h, followed by triple immunostaining and flow cytometric analysis for the expressions of CD14, TIM-3, and IL-12. PBMC were first gated on monocytic populations, and then further gated on CD14^+^ M/M_Ø_ (R3). Representative dot plots from a healthy subject demonstrate the relationship of Tim-3 and IL-12 expression and their response to β-gal or HCV core treatment. Summary data from 8 healthy subjects indicates the effect of HCV core on Tim-3 expression and IL-12 production in primary CD14^+^ M/M_Ø_. Mean values are expressed as percentage of the indicated population. Error bars represent the SD of the mean. The *p* value (**<0.01; ***<0.001) is denoted above the group of study subjects. B) Tim-3 expression and IL-12 production in THP-1 cells 72 h after HCV core or β-gal treatment followed by flow cytometric analysis. The histogram of Tim-3 or IL-12 expression is over-layed upon isotype control and different treatment. The data is reproducible in repeated experiments. C) Tim-3 (left panel) and IL-12 (right panel) expression in purified CD14^+^ M/M_Ø_ with no stimulation (blue line), LPS/R848/β-gal treatment (green line), or LPS/R848/core treatment (red line).

Previous studies suggest that Tim-3, PD-1, and SOCS-1 pathways are involved in negative regulation of T and B lymphocyte functions during HCV infection [10]–[11], [21]–[28]. Additionally, PD-1 and SOCS-1 has been demonstrated to negatively regulate IL-12 production in murine [29]–[32] as well as in human M/M_Ø_ during HCV infection or by HCV core treatment [33]–[34]. As such, we speculated that Tim-3 might also be involved in negative regulation of M/M_Ø_ function following exposure to HCV core protein. To examine this possibility, healthy PBMC were stimulated with LPS/R848 in the presence of HCV core or β-gal control for 18 h, and then subjected to analysis of Tim-3 and IL-12 expressions by flow cytometry. Fig. 4A shows the gating strategy in dot plots from a representative experiment and summary data from 8 subjects for analysis of Tim-3 versus IL-12 expressions in CD14^+^ M/M_Ø_ with no stimulation, LPS/R848/β-gal, and LPS/R848/core stimulation. Indeed, compared to β-gal treatment, HCV core protein up-regulates Tim-3 expression and down-regulates IL-12 production in CD14^+^ M/M_Ø_.

Up-regulation of Tim-3 in PBMC treated with HCV core might be secondary to stimulation by cytokines secreted from other immune cell populations rather than by a direct antigenic effect on M/M_Ø._ To exclude this possibility, we also stimulated a human monocytic cell line, THP-1, with LPS/R848 in the presence of HCV core or β-gal for 48 h and 72 h, and then detected Tim-3 and IL-12 expressions by flow cytometry. As shown in Fig. 4B, Tim-3 expression is significantly up-regulated, while IL-12 production is moderately down-regulated by HCV core treatment at 48 h (data not shown); and this dysregulation by HCV core protein is clearly observed at 72 h when compared to THP-1 cells treated with β-gal control protein. These data is reproducible in purified M/M_Ø_ treated with LPS and core for 18 h, in that i) Tim-3 is highly expressed on resting M/M_Ø_ with little if any IL-12 production; ii) Tim-3 significantly declines upon TLR stimulation that is accompanied by increased IL-12 expression; and iii) Tim-3 is moderately up-regulated along with IL-12 down-regulated by HCV core protein (Fig. 4C). Taken together, these results suggest that HCV core enhances Tim-3 expression on both primary CD14^+^ M/M_Ø_ and THP-1 cells, and inhibits IL-12 expression that is primarily produced by Tim-3 negative M/M_Ø_.

### HCV core mimics C1q to regulate M/M_Ø_ functions through interaction with gC1qR

**Figure 5:**
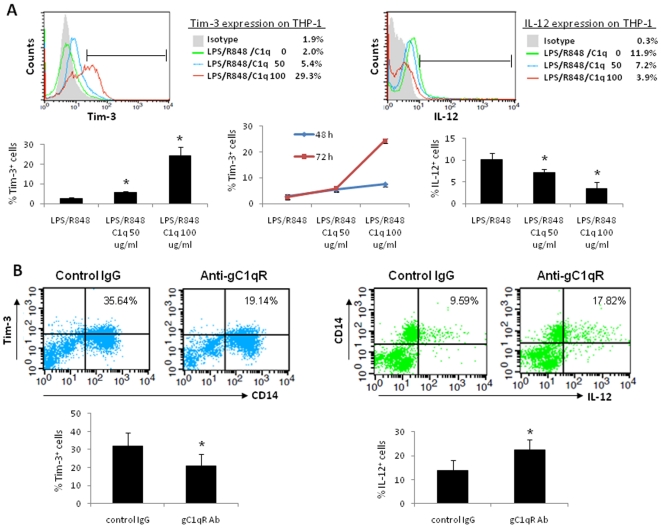
HCV core mimics C1q function in regulation of Tim-3 and IL-12 expression through interaction with gC1qR. A) Tim-3 and IL-12 expression in THP-1 cells treated with C1q (0, 50, and 100 µg/ml) for 48∼72 h. Representative histogram over-layed at 72 h is shown above, and summary data from 3 independent experiments is shown below. B) Regulation of Tim-3 and IL-12 expression in M/M_Ø_ by HCV core protein in a gC1qR-dependent manner. PBMC were pre-incubated with anti-gC1qR or control serum overnight, followed by the stimulation with LPS/R848 in the presence of HCV core protein for 18 h. Cell surface Tim-3 and intracellular IL-12 expression was detected by flow cytometry. Data are reproducible in 6 independent experiments.

We have previously demonstrated that HCV core protein differentially regulates T and B lymphocytes and inhibits IL-12 production through interaction with a complement receptor, gC1qR, expressed on these immune cells [23]–[28], [33]–[41]. gC1qR is an immunoreceptor initially identified by its ability to bind the globular head of C1q – the first component of the C1 complex in the complement system, which plays a crucial role in innate immunity against microbial antigens circulating in the blood of the infected host [42]. Engagement of C1q with gC1qR leads to multiple cellular activities including immunosuppression [42]. To determine whether HCV core protein mimics C1q function on innate immune cells through interaction with gC1qR, we treated THP-1 cells with gC1qR's natural ligand, C1q (0, 50, and 100 µg/ml), and LPS/R848 for 48∼72 h, followed by examination of the expressions of Tim-3 and IL-12 by flow cytometry. As shown in Fig. 5A, Tim-3 is up-regulated, while IL-12 is inhibited, by C1q in a dose-dependent manner. These results are reproducible in primary M/M_Ø_ treated with C1q for 18 h (data not shown), suggesting that HCV core mimics the function of C1q in inhibiting M/M_Ø_ IL-12 production by regulation of Tim-3 expression.

To determine whether HCV core protein truly alters gC1qR signaling to disrupt host immunity, we pre-incubated primary M/M_Ø_ with HCV core protein in the presence of anti-gC1qR antibody or control serum overnight, followed by LPS/R848 stimulation for 18 h, and then examined Tim-3/IL-12 expressions by flow cytometry. As shown in Fig. 5B, the core-mediated dysregulation of Tim-3/IL-12 is abrogated by antagonistic anti-gC1qR, but not by the control antibody, suggesting that HCV core up-regulates Tim-3 and down-regulates IL-12 expression through interaction with gC1qR on M/M_Ø_.

### Blocking the Tim-3 pathway suppresses PD-1 expression and enhances IL-12 production in CD14^+^ M/M_Ø_

**Figure 6:**
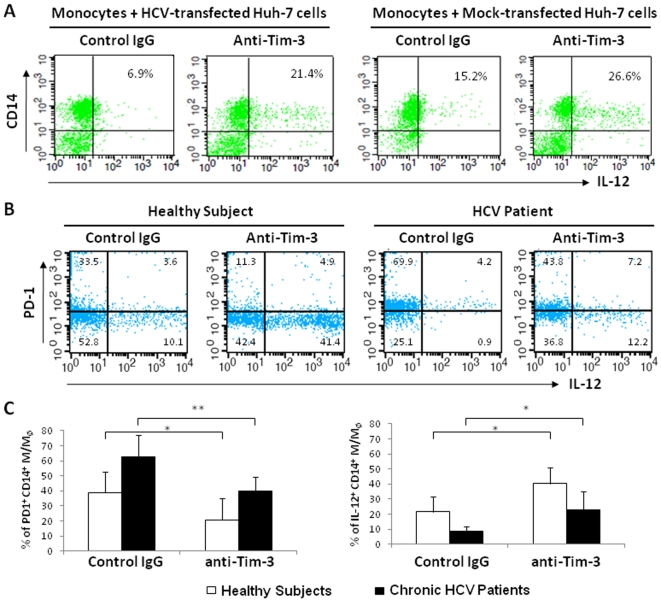
Tim-3 blockade significantly improves IL-12 production and reduces PD-1 expression on CD14^+^ M/M_Ø_. A) Purified M/M_Ø_ was incubated with Huh-7 cells in a ratio of 10∶1 at 48 h after HCV or mock transfection. Anti-Tim-3 or control IgG was added into the co-culture system at the same time and incubated overnight, followed by stimulation with LPS/R848 for another 18 h. IL-12 expression in CD14^+^ M/M_Ø_ was detected by flow cytometry. B) Representative flow cytometric dot plots from one healthy subject and one HCV-infected individual showing the effect of Tim-3 blocking on PD-1 and IL-12 expressions in CD14^+^ M/M_Ø_. Gating strategy for identifying CD14^+^ M/M_Ø_ subsets is the same as in Fig. 4A. C) PBMC from healthy subjects (n = 6) and chronically HCV-infected individuals (n = 6) were pre-incubated with anti-Tim-3 or control IgG antibodies 48 h, followed by stimulation with LPS/R848 for 18 h. PD-1 and IL-12 expressions were detected by flow cytometry. The percentages of PD-1^+^ CD14^+^ M/M_Ø_ or IL-12^+^ CD14^+^ M/M_Ø_ of healthy subjects as well as chronically HCV-infected individuals are shown. The *p* value (*<0.05, **<0.01) was denoted above the group of study subjects.

Based on the observations that Tim-3 is over-expressed on M/M_Ø_ that is associated with impaired IL-12 production in chronic HCV infection, and that HCV (core) contributes to the induction of Tim-3 and inhibition of IL-12 expression in M/M_Ø_, we next sought to examine the effect of Tim-3 blockade on M/M_Ø_ IL-12 production. We first employed the HCV-transfected Huh-7 cell culture system by adding anti-Tim-3 or control IgG and the purified M/M_Ø_ into 48 h-HCV− or mock-transfected Huh-7 cells overnight, followed by LPS/R848 stimulation for another 18 h. As shown in Fig. 6A, baseline IL-12 expression is relatively low in M/M_Ø_ incubated with HCV^+^ Huh-7 versus HCV^−^ Huh-7 cells and stimulated with LPS/R848/IgG for 18 h, confirming the data above that HCV exposure inhibits IL-12 expression (Fig. 3D). Nevertheless, IL-12 expression in CD14^+^ M/M_Ø_ is boosted by blocking Tim-3 signaling in the setting of co-culture with either HCV-transfected or mock-transfected hepatocytes.

We and others have recently found that PD-1 is involved in negative regulation of IL-12 production by M/M_Ø_ [29]–[30], [33]–[34]. Additionally, while Tim-3 and PD-1 could be single expressed on distinct M/M_Ø_, they are also duly expressed on certain populations of CD14^+^ M/M_Ø_, although the expression pattern and dynamics of these two negative regulatory molecules are quite different in innate immune cells (Zhang et al, under revision in JLB). To delineate any possible relationship between Tim-3 and PD-1 pathway in human M/M_Ø_ as an underlying mechanism for HCV-mediated IL-12 inhibition, we tested whether blocking the Tim-3 pathway could affect PD-1 expression and restore M/M_Ø_ function in chronically HCV-infected individuals. To this end, PBMC from 6 healthy subjects and 6 chronic HCV patients were incubated with anti-Tim-3 or control IgG antibody 48 h, followed by LPS/R848 stimulation for additional 18 h. Fig. 6B shows representative flow cytometric dot plots of a healthy and HCV subject measuring PD-1 surface expression and intracellular IL-12 production in CD14^+^ M/M_Ø_, and Fig. 6C summarizes the data from 6 subjects in each group. As we expected based on their cell surface expression patterns, the expression of PD-1 on M/M_Ø_ of healthy as well as HCV-infected subjects is significantly reduced by the blockade of Tim-3 pathway. Furthermore, Tim-3 blocking significantly improves IL-12 production by CD14^+^ M/M_Ø_ from both healthy and HCV-infected subjects when compared with the control IgG. *Vice versa*, blockade of the PD-1 pathway by PD-L1 antibody inhibits Tim-3 expression and improves IL-12 expression in M/M_Ø_ from HCV patients (Fig.S2). Collectively, TIM-3 signaling appears to crosstalk with the PD-1 pathway in regulation of IL-12 expression by M/M_Ø_ during HCV infection.

### Tim-3 blockade down-regulates HCV core-mediated SOCS-1 expression and up-regulates STAT-1 phosphorylation in human M/M_Ø_

**Figure 7:**
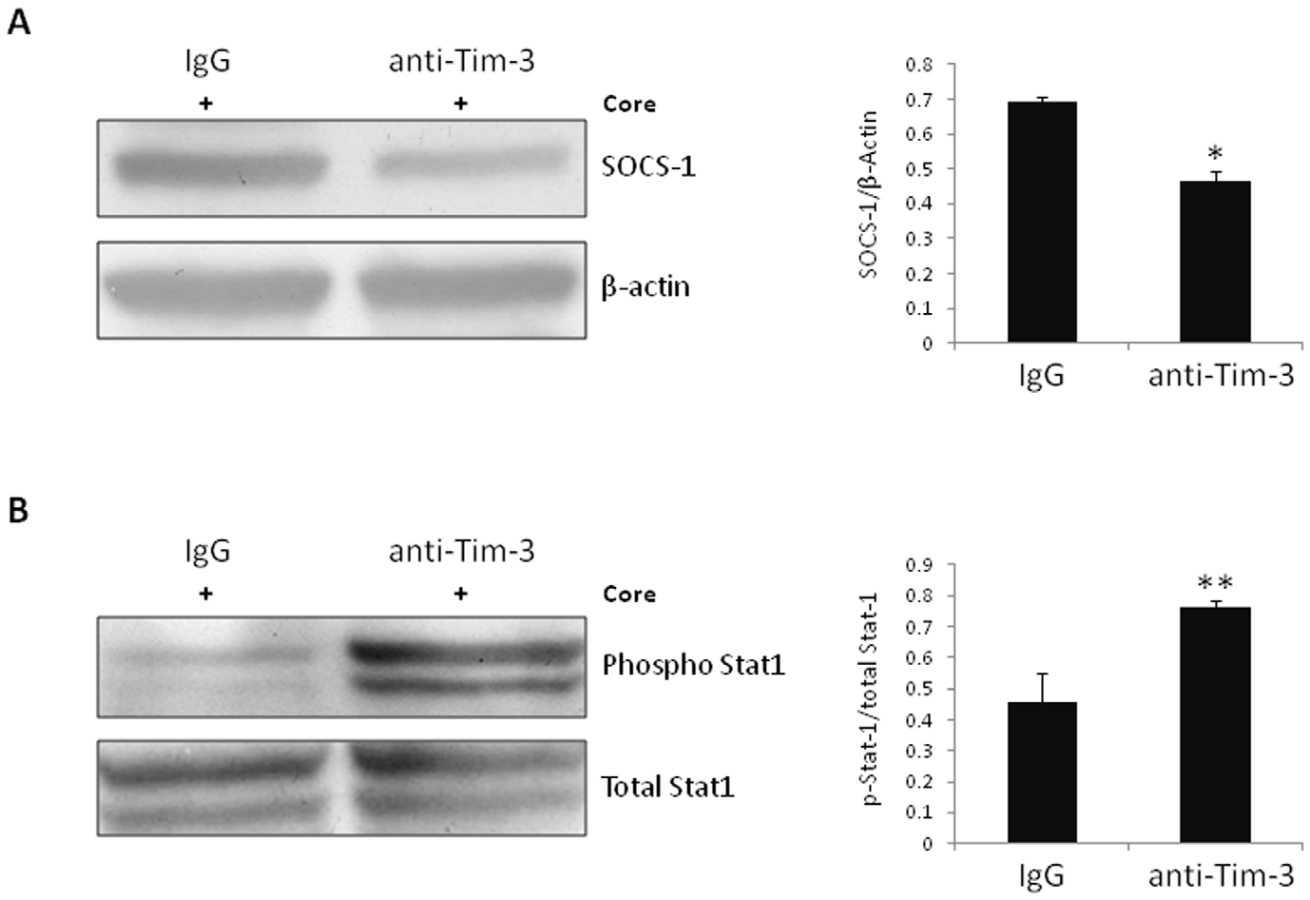
Tim-3 blockade down-regulates HCV core-mediated SOCS-1 expression and up-regulates STAT-1 phosphorylation in primary M/M_Ø_. Purified M/M_Ø_ was treated with Tim-3 antibody or control IgG overnight, followed by HCV core and LPS/R848 stimulation for 48 h. A) SOCS-1 or B) phospho-STAT-1 was detected by immunoblotting. Representative Western blot imaging is shown in the left, and summary of densitometry data with statistical analysis (*p<0.05, **p<0.01) from three independent experiments are shown on the right.

We have previously found that HCV core-induced differential regulation of T and B lymphocyte responses and inhibition of IL-12 expression in M/M_Ø_ is mediated by regulation of JAK/STAT signaling through induction of SOCS-1, which is a negative immunomodulator upstream of the JAK/STAT pathway [23]–[28], [33]–[41]. Since blockade of Tim-3 signaling enhances the function of CD14^+^ M/M_Ø_, we next explored the relationship between Tim-3 and SOCS-1 and whether Tim-3 also negatively modulates TLR-stimulated M/M_Ø_ IL-12 production through the JAK/STAT pathway. To this end, purified healthy M/M_Ø_ were incubated with anti-Tim-3 or control IgG overnight, followed by HCV core/LPS/R848 stimulation for 48 h. Cell lysates from these treated cells were immunoblotted to detect SOCS-1 and phosphorylated STAT-1 protein. β-actin and total STAT-1 were detected to serve as loading controls. As shown in Fig. 7A, Tim-3 blocking significantly down-regulates HCV core-induced SOCS-1 gene expression in M/M_Ø_ compared with cells treated with control IgG. In contrast to this decreased SOCS-1 expression, Tim-3 blockade rescues the HCV core-mediated inhibition of STAT-1 phosphorylation in M/M_Ø_ compared to those treated with control IgG (Fig. 7B). The statistical analysis of densitometry data from multiple experiments is significant, as summarized in the bar figures. SOCS-1 and JAK/STAT signaling pathways thus may be involved in Tim-3-mediated negative regulation of M/M_Ø_ function.

### Silencing SOCS-1 gene expression decreases Tim-3 expression and improves IL-12 production in THP-1 cells

**Figure 8:**
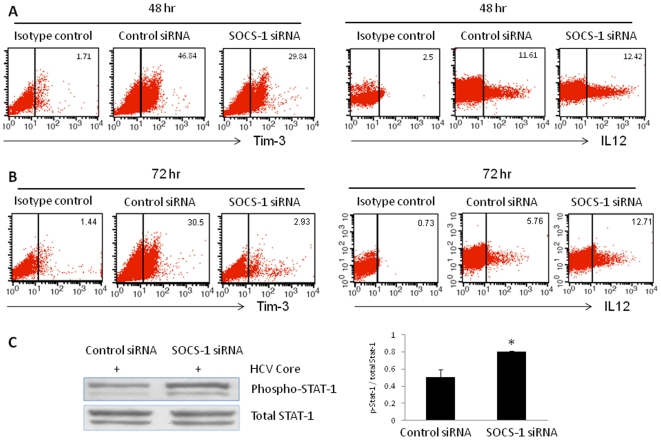
SOCS-1 silencing regulates HCV core-mediated Tim-3 expression, IL-12 production, and STAT-1 phosphorylation in THP-1 Cells. A) and B) THP-1 cells were transfected with SOCS-1 siRNA or control siRNA, followed by HCV core and LPS/R848 stimulation for another 48 and 72 h, then subjected for the expression of Tim-3 and IL-12 analysis by flow cytometry. C) The same cells were also subject to Western blot for detection of STAT-1 phosphorylation. The figures show one representative blot and summary densitometry data of 3 repeated experiments.

To further characterize the relationship between Tim-3 and SOCS-1 in negative regulation of TLR/core-mediated IL-12 regulation, we silenced SOCS-1 gene expression in THP-1 cells by transfection of a small interfering RNA (siRNA) specific for SOCS-1, followed by the treatment of HCV core and LPS/R848 for 48 h and 72 h. Compared with the control siRNA, THP-1 cells transfected with SOCS-1 siRNA exhibit significantly inhibited expression of SOCS-1 protein at both 48 and 72 h after transfection [34]. We measured Tim-3 expression and IL-12 production in THP-1 cells after SOCS-1 siRNA transfection and LPS/R848/core treatment for 48 h and 72 h. As shown in Fig. 8A and B, compared with control siRNA, Tim-3 expression is significantly suppressed on SOCS-1 siRNA-transfected THP-1 cells at both time points, but more significantly at 72 h after transfection. Functionally, transfection of SOCS-1 siRNA improved HCV core-suppressed IL-12 expression, especially at 72 h after transfection of SOCS-1 siRNA versus control siRNA. Similar to the reactivation of HCV core-mediated inhibition of STAT-1 phosphorylation by Tim-3 blocking (Fig. 7B), silencing SOCS-1 also rescued HCV core-induced STAT-1 dephosphorylation (Fig. 8C). Collectively, these data suggest that Tim-3 negatively regulates M/M_Ø_ IL-12 expression by crosstalk with other inhibitory molecules, including PD-1 and SOCS-1, through limiting STAT-1 phosphorylation.

## Discussion

Tim-3 is a newly identified inhibitory molecule that is up-regulated on dysfunctional T cells during chronic viral infections [7]–[10]. The expression and function of Tim-3 on human innate immune responses during HCV infection, however, remains unknown. Here, we report that 1) Tim-3 is constitutively expressed on resting M/M_Ø_, which have little IL-12 expression; upon TLR stimulation, Tim-3 expression is significantly reduced that accompanied by increased IL-12 production. 2) Tim-3 expression on un-stimulated and TLR-stimulated M/M_Ø_ in chronically HCV-infected individuals is significantly higher than healthy subjects, and this up-regulation can be recapitulated on healthy M/M_Ø_ exposure to HCV-expressing hepatocytes or HCV core protein. 3) Increased Tim-3 expression consistently correlates with decreased IL-12 expression by M/M_Ø_, an effect that mimics C1q function and is reversible by blocking the HCV core/gC1qR interaction. 4) Most importantly, the TLR-mediated IL-12 expression that occurs primarily in Tim-3 negative M/M_Ø_ in both healthy and HCV-infected patients could be improved by blocking the Tim-3 pathway. 5) Our findings also shed light on potential mechanisms by which Tim-3 suppresses M/M_Ø_ function by crosstalk with other negative immumodulators, including PD-1 and SOCS-1, and through altering JAK/STAT signaling.

The TIM gene family consists of eight members (Tim-1 through Tim-8) on mouse chromosome 11B1.1, and three members (Tim-1, 3, 4) on human chromosome 5q33.2, a chromosomal region that has been shown linked with asthma, allergy, and other autoimmune disorders [43]–[44]. All TIM-family proteins share a common architecture in which the extracellular region possesses a membrane-distal IgV domain and a membrane-proximal mucin domain [45]–[47]. Tim-3 plays an important role in the immune regulation of autoimmune diseases as well as viral infections and has been described as a bi-modal immune regulator in different disease scenarios [48]. In autoimmunity, loss of Tim-3 leads to excessive expansion of auto-reactive T cells [5], [49]. In chronic HCV, HIV, and HSV infections, in contrast, sustained Tim-3 expression on CD4^+^ and CD8^+^ T cells leads to T cell exhaustion [7]–[10], [50]. Gal-9 was identified as a ligand for Tim-3, and engagement of the Tim-3 IgV domain by gal-9 is important for termination of T cell responses and induction of T cell apoptosis as well as differentiation of regulatory T cells (Treg) expressing FoxP3 [11], [51], [52].

In addition to T lymphocytes, we now know that Tim-3 is also expressed by other immune cells, including natural killer cells (NK), dendritic cells (DC), monocytes and mast cells, and appears to have a much broader distribution and more complex role than previously thought [14], [53]–[55]. In the present study, Tim-3 was found constitutively expressed on naïve M/M_Ø_, functioning as a cap or brake to block pro-inflammatory cytokine IL-12 production. Tim-3 expression was significantly reduced, accompanying with enhanced IL-12 production following TLR stimulation. This is quite different from Tim-3 expression pattern on T cells, which show a very low level on naïve CD4^+^ and CD8^+^ T lymphocytes, increase level along with T cell activation (CD69 expression) by anti-CD3/CD28 stimulation (Fig.S3), although Tim-3 plays inhibitory role in both innate and adaptive immune cells (functioning as a cap in M/M_Ø_, while working in a feedback mechanism in T cells). Notably, our results demonstrate an expression pattern and functionality of Tim-3 that differs somewhat from the albeit limited human studies presented by Anderson AC et al, reporting that Tim-3 is primarily expressed on DCs and synergizes TLR to promote inflammation and serving opposite roles in the innate and adaptive immune systems [14]. This disparity is possibly due to the differences in the stimulation methods, the cell differential status, and the time-points chosen to evaluate the Tim-3 expression on human M/M_Ø_ in different laboratory. Our studies of Tim-3 expressions on M/M_Ø_ from both healthy and HCV-infected individuals show the same dynamic pattern suggest that it is not due to the difference of disease models. Our present data demonstrate that blockade of Tim-3 signaling significantly boosts LPS-mediated IL-12 production, which provides a firm demonstration that Tim-3 functions as a brake rather than a promoter in innate immune responses. This Tim-3 negative regulatory effect on innate immunity has also been reported by other investigators [12]–[13]. Notably, blocking Tim-3 in the absence of LPS stimulation does not alter IL-12 expression (Zhang et al, under review in JLB). This brake is at least transiently released following TLR activation, and its release without providing acceleration (TLR stimulation) has no consequence; whereas releasing Tim-3 and providing the necessary stimulation results in enhanced M/MØ function. We thus propose Tim-3 may indeed be important in permitting inflammation *per se* but does so by a decrease in its expression that permits TLR signaling to drive inflammatory responses.

Our observation that M/M_Ø_ from chronically HCV-infected subjects exhibit higher levels of Tim-3 expression and lower levels of IL-12 expression than healthy subjects implies a direct role for the HCV in the up-regulation of Tim-3 expression and functional impairment of M/M_Ø_. Notably, we simultaneously analyzed the expressions of IL-12, IL-6, IL-10, and TNF-α in CD14^+^ M/M_Ø_ from multiple HCV-infected individuals and healthy subjects by flow cytometry and found that IL-12 is selectively suppressed during chronic HCV infection in M/M_Ø_ in response to *ex vivo* LPS/R848 stimulation [33]. Additionally, to determine whether M/M_Ø_ from HCV-infected individuals fail to respond to these TLR ligands *ex vivo* because they lack the ability to sense pathogens, we also analyzed the expression of TLR4 and TLR7 in M/M_Ø_ from HCV-infected versus healthy subjects. To our surprise, TLR4 was found to be expressed high, but not low, on M/M_Ø_ from HCV-infected versus uninfected subjects, while TLR7 was highly detected, with no difference, in both HCV-infected and healthy subjects, signaling that the problem may lie at the downstream signaling pathways [33]. Multiple HCV antigens have been noted to be immunomodulatory and may be contributing to these findings, among which HCV core protein has a distinct immunosuppressive capacity and has been shown by us to inhibit IL-12 production in human M/M_Ø_ by yet unclear mechanisms [34], [40]. In the present study, we demonstrate that Tim-3 expression can be up-regulated by HCV core protein, accompanied by a down-regulation of IL-12 expression, in both primary healthy CD14^+^ M/M_Ø_ and THP-1 cells. IL-12 is mainly produced by Tim-3 negative M/M_Ø_, suggesting that high Tim-3 expression may identify a subset of functionally naïve or impaired M/M_Ø_ with limited IL-12 expression. This complements and integrates previous studies that have identified an important role for HCV core in immunoregulation [37], [56], and Tim-3 seems likely to be one of many molecules that mediate HCV core functionality. Certainly other HCV antigens may contribute to this dysregulation as well, and this deserves further investigation.

We have previously demonstrated that HCV core differentially regulates T and B lymphocyte functions and inhibits IL-12 production through interaction with gC1qR expressed on immune cells [23]–[38], [33]–[41]. gC1qR is a multifunctional protein that interacts with multiple ligands, including many derived from pathogenic organisms [42], such as HIV-Tat and Rev, EBV EBNA-1, HSV-ORF P, adenovirus core protein-V, *L. monocytogenes*-InlB, and S. aureus-protein A. These pilot studies suggest that microorganisms may capitalize gC1qR as a shared mechanism of immune evasion, and thus the role of gC1qR in the pathogenesis of these pathogens has been the subject of further investigation. In this study, we demonstrate that C1q, the natural ligand of gC1qR, can dose-dependent up-regulate Tim-3 and inhibit IL-12 expression in a manner similar to HCV core protein, which has been shown to bind gC1qR as one of its mechanisms of immunomodulation [35], [39]. Importantly, blocking gC1qR abrogates HCV core-induced Tim-3/IL-12 dysregulation. To our knowledge, this is the first demonstration that HCV core protein mimics the function of complement C1q, at least in part, in inhibition of IL-12 expression by up-regulation of Tim-3 through interaction with gC1qR.

The mechanisms of HCV-mediated Tim-3 up-regulation probably involve in the following two aspects: i) HCV may increase Tim-3 gene transcription or translation; ii) HCV may decrease or slowdown Tim-3 protein degradation. HCV inhibits monocyte activation might be a cause for Tim-3 elevation detected in both HCV-infected patients or co-cultured with HCV-expressing hepatocytes, rather than HCV-induced Tim-3 causes cell inactivation; however, blocking Tim-3 signaling significantly improved IL-12 production, suggesting the causing effect of Tim-3 in control of IL-12 expression. Notably, improvement of cell function by blocking Tim-3 pathway is observed in the absence of HCV core, and this phenomenon has also been demonstrated by us for other inhibitory molecules such as PD-1 and SOCS-1, in M/M_Ø_ as well as in T cells [26], [34]. These results indicate that Tim-3, PD-1, and SOCS-1 inhibitory molecules are pre-existing and function in a network in cell negative regulation. It seems plausible that HCV, in order to survival or persistence *in vivo*, may exploit this pre-existing cell mechanism to impair host immunity, i.e., HCV (core) is taking advantage of the intrinsic mechanism by magnification of these negative signaling molecules, breaking the balance to positive co-stimulatory molecules, for its own benefit of survival in the host, thus leading to viral persistence.

In this study, blockade of Tim-3 pathway was found to enhance IL-12 production in M/M_Ø_ of healthy and HCV-infected subjects, suggesting that the Tim-3 pathway plays a crucial role in suppressing M/M_Ø_ functions. Additionally, IL-12 expression is affected not only by Tim-3, but by other negative immunomodulators, such as PD-1 and SOCS-1 [29]–[34]. As we show in this study, blockade of the Tim-3 pathway suppresses PD-1 expression as well as HCV core-mediated PD-1/SOCS-1 expression and improves STAT-1 phosphorylation in primary M/M_Ø_, while blocking PD-1 signaling or silencing SOCS-1 gene expression also decreases Tim-3 expression and enhances IL-12 secretion and STAT-1 phosphorylation. This implies potential crosslink amongst these molecules in negatively regulating innate immunity during chronic HCV infection. These observations support a plausible notion that Tim-3, PD-1 and SOCS-1, while seemingly regulating cell signaling at different levels, are actually associated or linked in an inhibitory circuit or form a cluster to prevent ubiquitin-degradation, and exert an integrated role in suppressing cell signal transduction. Thus, blockade the signaling of one molecule leads to a change (either decreased degradation or increased transcription/translation) in the others within cells to improve the IL-12 expression, which has been consistently demonstrated in this and our other studies.

**Figure 9:**
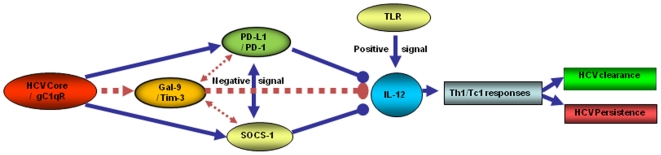
A Model for the HCV core/gC1qR-induced negative signaling (Tim-3/PD-1/SOCS-1) molecules in regulation of IL-12 and Th1/Tc1 responses during HCV infection. We have previously shown that HCV core/gC1qR interaction up-regulates PD-1 and SOCS-1 negative signaling molecules, leading to suppression of TLR-mediated IL-12 production. In this study, we further demonstrated that the Tim-3 inhibitory pathway is involved in the HCV core/gC1qR-induced inhibition of IL-12 expression by M/M_Ф_ during HCV infection. Specifically, we found that Tim-3 can be up-regulated by HCV core/gC1qR interaction, which in turn, inhibits TLR-mediated IL-12 production. We also found that Tim-3 can crosstalk with other inhibitory molecules such as PD-1 and SOCS-1 to coordinately inhibit TLR-mediated IL-12 signaling pathways during HCV infection. We conclude that HCV-mediated innate immune dysregulation (impaired M/M_Ф_ IL-12 production) may ultimately lead to adaptive Th1/Tc1 dysfunction, and thus, HCV persistence.

Studies on the pathogenesis of HCV have been significantly advanced since the establishment of an *in vitro* cell culture model using Huh-7 hepatocytes-transfected with HCV-JFH-1 strain [19]–[20]. With the aid of Drs T. Wakita and T.J. Liang [19]–[20], we have successfully established an HCV-transfected Huh-7 model co-cultured with purified M/M_Ø_, a model which provides us with a unique cell culture system to study host cell interactions that can more closely mimic the *in vivo* setting. By employing this novel system in our present study, we have demonstrated that Tim-3 is up-regulated by HCV to inhibit IL-12 expression. Based on this and our previous studies, we propose a pathogenesis model (Fig. 9) in that HCV core protein secreted from HCV-infected hepatocytes binds to gC1qR displayed on macrophages, up-regulating Tim-3, PD-1, and SOCS-1 negative immunomodulators that can crosstalk each other and coordinately inhibit cell signaling transduction, resulting in an impaired innate immune response with a cytokine environment (deficient IL-12/TNFα/IFN-γ) that is permissive for suppression of adaptive immune responses in the liver so as to facilitate the establishment of persistent infection. Whether other structural or non-structural proteins in additional to HCV core protein contribute to Tim-3/IL-12 dysregulation is under investigation in our laboratory using this model system.

In conclusion, this study presents a novel and crucial role for Tim-3 as a negative regulator of M/M_Ø_ function in innate immune responses to HCV infection. The identification of Tim-3 expression and function in innate immune cells in a human disease model provides a new perspective to understanding the roles of this negative molecule as a potential target for immune therapy of chronic HCV infection.

## Materials and Methods

### Subjects

The study subjects composed of two populations, with the first group consisting of 21 chronically HCV-infected subjects. HCV genotype and viral load were performed by Lexington VAMC and all subjects were virologically and serologically positive for HCV, prior to the treatment with IFN/RBV (Table 1). The second group includes 12 healthy subjects who are negative for HBV, HCV, and HIV infections. Written informed consent was obtained from all participants, and the study was approved by an institutional review board at East Tennessee State University and James H. Quillen VA Medical Center (Johnson City, TN).

**Table 1:** Characteristics of subjects included in the study.

Subject	Age	Race	Gender	ALT/AST	Genotype	Viral Load (IU/ml)
1	59	Caucasian	M	78/61	1a	2,569,032
2	51	African-American	M	65/79	1a	8,476,003
3	52	Caucasian	M	73/55	1a	489,591
4	59	Caucasian	M	30/24	4b	887,509
5	48	Caucasian	M	65/55	1a	4,547,924
6	49	Caucasian	M	103/141	1a	2,298,550
7	63	Caucasian	M	99/69	3a	3,260,000
8	51	Caucasian	M	109/92	1a	3,653
9	35	Caucasian	M	34/43	2b	40,100,000
10	53	Caucasian	M	76/89	1a	1,890,00
11	50	Caucasian	M	163/137	3a	11,641,000
12	35	Caucasian	M	121/109	3a	4,190,619
13	51	Caucasian	M	99/103	1a	4,790,000
14	48	African-American	M	133/112	1a	422,263
15	53	Caucasian	M	73/59	1a	173,294
16	56	Caucasian	M	178/146	3a	1,360,000
17	52	Caucasian	M	109/135	3a	30,900,000
18	26	Caucasian	M	83/57	1a	1, 106,553
19	55	Caucasian	M	65/77	1a	69,300
20	49	African-American	M	112/134	1a	20,000 ,000
21	56	Caucasian	M	89/57	1a	5,206 ,273

### Cell isolation and culture

Human peripheral blood mononuclear cells (PBMC) were isolated from whole blood using Ficoll density gradient centrifugation (Atlanta biological, Lawrenceville, GA). Human primary M/M_Ø_ were isolated from buffy coats using Ficoll-Percoll gradients (GE Heathcare, Piscataway, NJ). PBMC and M/M_Ø_ were viably frozen in 30% fetal bovine serum (Life Technologies, Gaithersburg, MD), 10% dimethyl sulfoxide (DMSO) and 60% RPMI 1640 medium (Mediatech, Inc, Manassas, VA) in liquid nitrogen. For subsequent analysis, the frozen PBMC and M/M_Ø_ were thawed, washed and cultured with RPMI 1640, containing 10% fetal bovine serum (FBS, Life Technologies, Gaithersburg, MD), 100 µg/ml penicillin-streptomycin (Thermo Scientific, Logan, Utah), and 2 mM L-glutamine (Thermo Scientific, Logan, Utah). A human monocytic cell line, THP-1 cells, was obtained from ATCC (American Type Culture Collection, Manassas, VA) and cultured in RPMI 1640 (ATCC, Manassas, VA), containing 10% FBS, 100 µg/ml penicillin-streptomycin, and 0.05 mM β-mercaptoethanol (Sigma-Aldrich, St. Louis, MO) at 37°C with 5% CO_2_ atmosphere.

### Flow cytometry

PBMC or THP-1 cells were stimulated by 5 µg/ml of LPS and 5 µg/ml of R848 (InvivoGen, San Diego, CA - that can synergistically activate primary M/M_Ø_ to produce IL-12) in the presence or absence of 2 µg/ml of HCV core protein (ViroGen, Watertown, MA) or 50 and 100 µg/ml C1q (Quidel Corporation, San Diego, CA) for 18∼72 h depending on the experimental requirements as described in the result, followed by Brefeldin A (BioLegend, San Diego, CA) 6 h prior to harvest the cells for inhibiting cytokine secretion. Specific antibody direct conjugates for cell surface staining was carried out using Tim-3 (R&D, Minneapolis, MN), or PD-1 (BD Biosciences, San Jose, CA), and CD14 (Miltenyi Biotec Inc, Auburn CA), followed by intracellular staining for IL-12 (Miltenyi Biotec Inc). The intracellular cytokine staining was carried out using an Inside Stain kit (Miltenyi Biotec Inc). Isotype-matched control antibodies (BD Biosciences, San Jose, CA) were used to determine background levels of staining. The cells were analyzed on a FACSCalibur flow cytometry (BD, Franklin Lakes, NJ) and CELLQuest software.

### Huh-7 hepatocytes transfection by HCV-JFH-1 and co-culture with primary M/M_Ø_

HCV JFH-1 (kindly provided by Dr. T. Wakita, Department of Virology II, NIH of Japan through a MTA) was transfected into MAX Efficiency® DH5α™ Competent Cells, replicated, and purified by a plasmid miniprep kit (Invitrogen corporation, Carlsbad, CA). The purified DNA was linearized with XbaI (Promega Corporation. Madison, WI) and Mung Bean Nuclease (New England BiolabsIpswich, MA), and further purified with OriGene powerprepTM Express PCR purification kit (OriGene Technologies, Inc. Rockville, MD). Reverse transcription of the DNA into mRNA was carried out by a MEGA script® T7 Kit and purified by MEGAclearTM kit (Ambion,Inc Austin, TX) per manufactruer's instructions. 5×10^5^ Huh-7 hepatocytes (kindly provided by Dr. T.J.Liang, liver section, NIH/NIDDK) were transfected at 60∼70% confluent in a 6-well plate with 5 µg HCV mRNA using DMRIE-C reagents per company's protocol (invitrogen corporation, Carlsbad, CA). HCV antigen expression was examined at 24, 48, and 72 h after transfection by immunoflourescence using HCV core and NS5 antibodies (ViroGen, Watertown MA). HCV replication was also demonstrated by RT-PCR amplify HCV core mRNA from the supernatant of JFH-1-transfected Huh-7 culture, and by inoculation of uninfected Huh-7 cells with the 48 h supernatant of JFH-1-transfected Huh-7 culture. Mock-transfection (without HCV RNA) of Huh-7 cells was carried out in the same way as negative control. For the co-culture experiment, purified M/M_Ø_ were incubated with hepatocytes in a ratio of 10∶1 at 48 h after HCV/mock-transfection, and stimulated with or without LPS/R848 for another 6∼48 h. The expression of Tim-3 and IL-12 in M/M_Ø_ was examined by flow cytometry.

### Tim-3, PD-1, or gC1qR blockade

Healthy and/or HCV patients' PBMC or purified M/M_Ø_ were incubated with 10 µg/ml anti-TIM3 (R&D Systems) or 5 µg/ml anti-PDL-1 (eBioscience) or 1∶10 diluted antagonistic gC1qR antibody (generously provided by Dr. Y.S. Hahn, University of Virginia) or control IgG overnight, followed by stimulation with 2 µg/ml of HCV core protein (ViroGen, Watertown, MA), 5 µg/ml of LPS and 5 µg/ml of R848 for 24∼72 h, then subjected for flow cytometric detection of PD-1 or Tim-3 and IL12 expressions. Tim-3-blocked, purified monocytes treated as above were also lysed for Western blot detection of SOCS-1 (Millipore, Temecula, CA) and phospho-STAT-1 (Tyr 701, Cell Signaling Technology, Inc. Danvers, MA). β-actin and total STAT-1 serve as loading control.

### siRNA silencing of SOCS-1

3×10^5^ THP-1 cells were incubated with 60 pmols SOCS-1 siRNA duplex or control siRNA in 200 µl siRNA Transfection Medium in a 6-well plate per manufacturer's instruction (Santa Cruz Biotechnology, Santa Cruze, CA). Following 6 h of incubation at 37°C, normal growth medium containing 2× concentrations of normal serum and antibiotics was added. The transfected cells were stimulated with 5 µg/ml LPS and R848 in the presence of 2 µg/ml HCV core for 48∼72 hrs, followed by detection of SOCS-1/Tim-3/STAT-1/IL-12 as described above.

### Western blot

The purified M/M_Ø_ or THP-1 cells were treated as described in the Tim-3 blocking or SOCS-1 silencing experiments and the expression of SOCS-1 or phospho-STAT-1 were measured by Western blot. The primary M/M_Ø_ or THP-1 cells were lysed in 1×RIPA lysis buffer (Boston BioProducts Inc, Ashland, MA) supplied with protease inhibitors/phosphorylase inhibitors (Thermo Scientific, Rockford, IL) and EDTA on ice. Cell lysates were centrifuged for 15 min at 4°C and the protein concentrations were measured. Protein samples were thereafter combined with 4× Laemmli sample buffer (Boston BioProducts, Ashland, MA), denatured, and separated by SDS-PAGE. Following transfer to an Amersham Hybond-P membrane (GE Heathcare, Piscataway, NJ), the membrane was blocked and probed with anti-SOCS-1 (Millipore, Temecula, CA) or actin (Santa Cruz Biotechnology, Santa Cruze, CA) antibody at 4°C overnight. For detection of phospho-STAT-1 and total STAT-1, the membrane was probed with anti-Phospho-STAT-1 (Tyr701) or total STAT-1 antibody (Cell Signaling Technology, Inc, Danvers, MA). Finally, the membrane was incubated with a horseradish peroxidase-conjugated secondary antibody (Millipore, Temecula, CA) and developed by Amersham™ ECL Plus Western Blotting Detection Reagents (GE Healthcare Biosciences, Pittsburgh, PA) on Kodak X-OMAT-LS X-ray film (Sigma-Aldrich, St. Louis, MO).

### Statistical analysis

Study results are summarized for each group and results are expressed as the mean ± standard deviation (SD). Comparison between two groups is performed using multiple comparison testing—least significant difference or Turkey's procedure depending on the ANOVA F-test by SPSS 18 software. Barforonni correction is applied for those samples with multiple tests. Pair wise t-test is used to compare the significance of PD-1 and IL-12 expressions in Tim-3 blocking experiment. Correlations between TIM-3 expression and IL-12 production were analyzed by a Pearson Correlation program. Values of *p*<0.05 (*), *p*<0.01(**), and *p*<0.001 (***) were considered significant or very significant.

## Supporting Information

Figure S1 **Conformation of HCV replication in hepatocyte culture system.** A) Immunofluorescience staining of HCV NS5 protein in HCV-transfected Huh-7 versus mock-transfected Huh-7 cells at 48 h. B) RT-PCR of HCV core mRNA in the supernantant of HCV-transfected Huh-7 versus mock-transfected Huh-7 cells at 36 h. C) Immunofluorescience staining of HCV NS5 protein in Huh-7 cells infected by the supernatant of HCV-transfected Huh-7 cells at 48 h.
(TIF)

Figure S2 **Blockade of PD-1 signaling by anti-PDL-1 antibody decreases Tim-3 expression and improves IL-12 production by CD14^+^ M/M_Ø_.**
(TIF)

Figure S3 **Tim-3 and CD69 expressions on naïve and activated CD4^+^ and CD8^+^ T lymphocytes.** PBMC from 3 healthy subjects were stimulated with or without anti-CD3/CD28 for 24 h followed by flow cytometric analysis of Tim-3 and CD69 expressions on CD4^+^ and CD8^+^ T cells. Summary data of percentages of Tim-3^+^ or CD69^+^ cells in naïve versus activated CD8^+^ (A) or CD4^+^ (B) lymphocytes are shown, and the *p* value (**<0.01; ***<0.001) is denoted above the group of study subjects.
(TIF)

## References

[1] Shepard CW, Finelli L, Alter MJ (2005). Global epidemiology of hepatitis C virus infection.. Lancet Infect Dis.

[2] Kuchroo VK, Umetsu DT, DeKruyff RH, Freeman GJ (2003). The TIM gene family: emerging roles in immunity and disease.. Nat Rev Immunol.

[3] Khademi M, Illés Z, Gielen AW, Marta M, Takazawa N (2004). T Cell Ig- and mucin-domain-containing molecule-3 (TIM-3) and TIM-1 molecules are differentially expressed on human Th1 and Th2 cells and in cerebrospinal fluid-derived mononuclear cells in multiple sclerosis.. J Immunol.

[4] Zhu C, Anderson AC, Schubart A, Xiong H, Imitola J (2005). The Tim-3 ligand galectin-9 negatively regulates T helper type 1 immunity.. Nat Immunol.

[5] Koguchi K, Anderson DE, Yang L, O'Connor KC, Kuchroo VK (2006). Dysregulated T cell expression of TIM3 in multiple sclerosis.. J Exp Med.

[6] Hastings WD, Anderson DE, Kassam N, Koguchi K, Greenfield EA (2009). TIM-3 is expressed on activated human CD4+ T cells and regulates Th1 and Th17 cytokines.. Eur J Immunol.

[7] Jones RB, Ndhlovu LC, Barbour JD, Sheth PM, Jha AR (2008). Tim-3 expression defines a novel population of dysfunctional T cells with highly elevated frequencies in progressive HIV-1 infection.. J Exp Med.

[8] Vali B, Jones RB, Sakhdari A, Sheth PM, Clayton K (2010). HCV-specific T cells in HCV/HIV co-infection show elevated frequencies of dual Tim-3/PD-1 expression that correlate with liver disease progression.. Eur J Immunol.

[9] Golden-Mason L, Palmer BE, Kassam N, Townshend-Bulson L, Livingston S (2009). Negative immune regulator Tim-3 is overexpressed on T cells in hepatitis C virus infection and its blockade rescues dysfunctional CD4+ and CD8+ T cells.. J Virol.

[10] Jin HT, Anderson AC, Tan WG, West EE, Ha SJ (2010). Cooperation of Tim-3 and PD-1 in CD8 T-cell exhaustion during chronic viral infection.. PNAS.

[11] Mengshol JA, Golden-Mason L, Arikawa T, Smith M, Niki T (2010). A crucial role for kupffer cell-derived galectin-9 in regulation of T cell immunity in hepatitis C infection.. PLoS one.

[12] Monney L, Sabatos CA, Gaglia JL, Ryu A, Waldner H (2002). Th1-specific cell surface protein Tim-3 regulates macrophage activation and severity of an autoimmune disease.. Nature.

[13] Frisancho-Kiss S, Nyland JF, Davis SE, Barrett MA, Gatewood SJ (2006). Cutting edge: T cell Ig mucin-3 reduces inflammatory heart disease by increasing CTLA-4 during innate immunity.. J Immunol.

[14] Anderson AC, Anderson DE, Bregoli L, Hastings WD, Kassam N (2007). Promotion of tissue inflammation by the immune receptor Tim-3 expressed on innate immune cells.. Science.

[15] Nakayama M, Akiba H, Takeda K, Kojima Y, Hashiguchi M (2009). Tim-3 mediates phagocytosis of apoptotic cells and cross-presentation.. Blood.

[16] Dai SY, Nakagawa R, Itoh A, Murakami H, Kashio Y (2005). Galectin-9 induces maturation of human monocyte-derived dendritic cells.. J Immunol.

[17] Bohnenkamp HR, Papazisis KT, Burchell JM, Taylor-Papadimitriou J (2007). Synergism of Toll-like receptor-induced interleukin-12p70 secretion by monocyte-derived dendritic cells is mediated through p38 MAPK and lowers the threshold of T-helper cell type 1 responses.. Cellular immunology.

[18] Wenink MH, Santegoets KC, Broen JC, van Bon L, Abdollahi-Roodsaz S (2009). TLR2 promotes Th2/Th17 responses via TLR4 and TLR7/8 by abrogating the type I IFN amplification loop.. J Immunol.

[19] Wakita T (2009). Isolation of JFH-1 strain and development of an HCV infection system.. Methods Mol Biol.

[20] Kato T, Matsumura T, Heller T, Saito S, Sapp RK (2007). Production of infectious hepatitis C virus of various genotypes in cell cultures.. J Virol.

[21] Golden-Mason L, Palmer B, Klarquist J, Mengshol JA, Castelblanco N (2007). Upregulation of PD-1 expression on circulating and intrahepatic hepatitis C virus-specific CD8+ T cells associated with reversible immune dysfunction.. J Virol.

[22] Kasprowicz V, Schulze Zur Wiesch J, Kuntzen T, Nolan BE, Longworth S (2008). High level of PD-1 expression on hepatitis C virus (HCV)-specific CD8+ and CD4+ T cells during acute HCV infection, irrespective of clinical outcome.. J Virol.

[23] Yao ZQ, King E, Prayther D, Yin D, Moorman J (2007). T cell dysfunction by hepatitis C virus core protein involves PD-1/PDL-1 signaling.. Viral Immunol.

[24] Yao ZQ, Prayther D, Trabue C, Dong ZP, Moorman JP (2008). Differential regulation of SOCS-1 signaling in B and T lymphocytes by hepatitis C virus core protein.. Immunology.

[25] Moorman JP, Dong ZP, Ni L, Zhang CL, Frazier A (2009). Abnormal B lymphocyte activation associated with TALL-1 overexpression and SOCS-1 deregulation in chronic HCV infection.. Immunology.

[26] Frazier AD, Zhang CL, Ni L, Ma CJ, Zhang Y (2010). Program death-1 pathway affects suppressor of cytokine signaling-1 expression in T cells during hepatitis C infection.. Viral Immunology.

[27] Ni L, Ma CJ, Zhang Y, Nandakumar S, Zhang CL (2010). PD-1 modulates Regulatory T cells and suppresses T cell responses in HCV-associated Lymphoma.. Immunology & Cell Biology.

[28] Yao ZQ, Ni L, Zhang Y, Ma CJ, Zhang CL (2011). Differential Regulation of T and B lymphocytes by PD-1 and SOCS-1 signaling in Hepatitis C Virus-associated non-Hodgkin's Lymphoma.. Immunol Invest.

[29] Cho HY, Choi EK, Lee SW, Jung KO, Seo SK (2009). Programmed death-1 receptor negatively regulates LPS-mediated IL-12 production and differentiation of murine macrophage RAW264.7 cells.. Immunol Lett.

[30] Yao S, Wang S, Zhu Y, Luo L, Zhu G (2009). PD-1 on dendritic cells impedes innate immunity against bacterial infection.. Blood.

[31] Said EA, Dupuy FP, Trautmann L, Zhang Y, Shi Y (2010). Programmed death-1-induced interleukin-10 production by monocytes impairs CD4+ T cell activation during HIV infection.. Nat Med.

[32] Eyles JL, Metcalf D, Grusby MJ, Hilton DJ, Starr R (2002). Negative regulation of interleukin-12 signaling by suppressor of cytokine signaling-1.. J Biol Chem.

[33] Ma CJ, Ni L, Zhang Y, Zhang CL, Wu XY (2010). PD-1 negatively regulates IL-12 expression by limiting STAT-1 phosphorylation in monocytes/macrophages during chronic hepatitis C infection.. Immunology.

[34] Zhang Y, Ma CJ, Ni L, Zhang CL, Wu XY (2011). Crosstalk between PD-1 and SOCS-1 in HCV core-mediated IL-12 suppression.. J Immunol.

[35] Kittlesen DJ, Chianese-Bullick KA, Yao ZQ, Braciale TJ, Hahn YS (2000). Interaction between complement receptor gC1qR and hepatitis C virus core protein inhibits T lymphocyte proliferation.. J Clin Invest.

[36] Yao ZQ, Nguyen DT, Hiotellis AI, Hahn YS (2001). Hepatitis C virus core protein inhibits human T lymphocyte responses by a complement-dependent regulatory pathway.. J Immunol.

[37] Yao ZQ, Ray S, Eisen-Vandervelde A, Waggoner S, Hahn YS (2001). Hepatitis C virus: Immunosuppression by complement regulatory pathway.. Viral Immunology.

[38] Yao ZQ, Eisen-Vandervelde A, Ray S, Hahn YS (2003). HCV core/gC1qR interaction arrests T cell cycle progression through stabilization of the cell cycle inhibitor p27^kip1^.. Virology.

[39] Yao ZQ, Eisen-Vandervelde A, Waggoner SN, Cale EM, Hahn YS (2004). Direct binding of hepatitis C virus core to gC1qR on CD4+ and CD8+ T cells leads to impaired activation of Lck and Akt.. J Virol.

[40] Eisen-Vandervelde AL, Waggoner SN, Yao ZQ, Cale EM, Hahn CS (2004). Hepatitis C virus core selectively suppresses interleukin-12 synthesis in human macrophages by interfering with AP-1 activation.. J Bio Chem.

[41] Moorman JP, Zhang CL, Ni L, Ma CJ, Zhang Y (2011). Impaired hepatitis B vaccine responses during chronic hepatitis C infection: involvement of the PD-1 pathway in regulating CD4^+^ T cell responses.. Vaccine.

[42] Ghebrehiwet B, Lim B, Kumar R, Feng X, Peerchke EIB (2001). gC1qR/p33, a member of a new class of multifunctional and multicompartmental cellular proteins, is involved in inflammation and infection.. Immunol Rev.

[43] Rodriguez-Manzanet R, DeKruyff R, Kuchroo VK, Umetsu DT (2009). The costimulatory role of TIM molecules.. Immunol Rev.

[44] Meyers JH, Sabatos CA, Chakravarti S, Kuchroo VK (2005). The TIM gene family regulates autoimmune and allergic diseases.. Trends Mol Med.

[45] Cao E, Zang X, Ramagopal UA, Mukhopadhaya A, Fedorov A (2007). T cell immunoglobulin mucin-3 crystal structure reveals a galectin-9-independent ligand-binding surface.. Immunity.

[46] Santiago C, Ballesteros A, Martínez-Muñoz L, Mellado M, Kaplan GG (2007). Structures of T cell immunoglobulin mucin protein 4 show a metal-ion-dependent ligand binding site where phosphatidylserine binds.. Immunity.

[47] Santiago C, Ballesteros A, Tami C, Martínez-Muñoz L, Kaplan GG (2007). Structures of T cell immunoglobulin mucin receptors 1 and 2 reveal mechanisms for regulation of immune responses by the TIM receptor family.. Immunity.

[48] Hafler DA, Kuchroo V (2008). TIMs: central regulators of immune responses.. J Exp Med.

[49] Yang L, Anderson DE, Kuchroo J, Hafler DA (2008). Lack of TIM-3 immunoregulation in multiple sclerosis.. J Immunol.

[50] Sehrawat S, Reddy PB, Rajasagi N, Suryawanshi A, Hirashima M (2010). Galectin-9/TIM-3 interaction regulates virus-specific primary and memory CD8 T cell response.. PLoS Pathog.

[51] Rabinovich GA, Toscano MA (2009). Turning ‘sweet’ on immunity: galectinglycan interactions in immune tolerance and inflammation.. Nat Rev Immunol.

[52] Seki M, Oomizu S, Sakata KM, Sakata A, Arikawa T (2008). Galectin-9 suppresses the generation of Th17, promotes the induction of regulatory T cells, and regulates experimental autoimmune arthritis.. Clin Immuno.

[53] Ju Y, Hou N, Meng J, Wang XY, Zhang XN (2010). T cell immunoglobulin- and mucin-domain-containing molecule-3 (Tim-3) mediates natural killer cell suppression in chronic hepatitis B.. J Hepatol.

[54] Kuchroo VK, Dardalhon V, Xiao S, Anderson AC (2008). New roles for TIM family members in immune regulation.. Nat Rev Immunol.

[55] Wiener Z, Kohalmi B, Pocza P, Jeager J, Tolgyesi G (2007). TIM-3 is expressed in melanoma cells and is upregulated in TGF-beta stimulated mast cells.. J Invest Dermatol.

[56] Eisen-Vandervelde A, Yao ZQ, Hahn YS (2004). The molecular basis of HCV-mediated immune dysregulation.. Clinical Immunology.

