# Supplementary figures and images for: Tim-3 Negatively Regulates IL-12 Expression by Monocytes in HCV Infection
Source: PLoS One. 2011 May 26;6(5):e19664. doi: 10.1371/journal.pone.0019664 (PMC3102652; doi:10.1371/journal.pone.0019664)

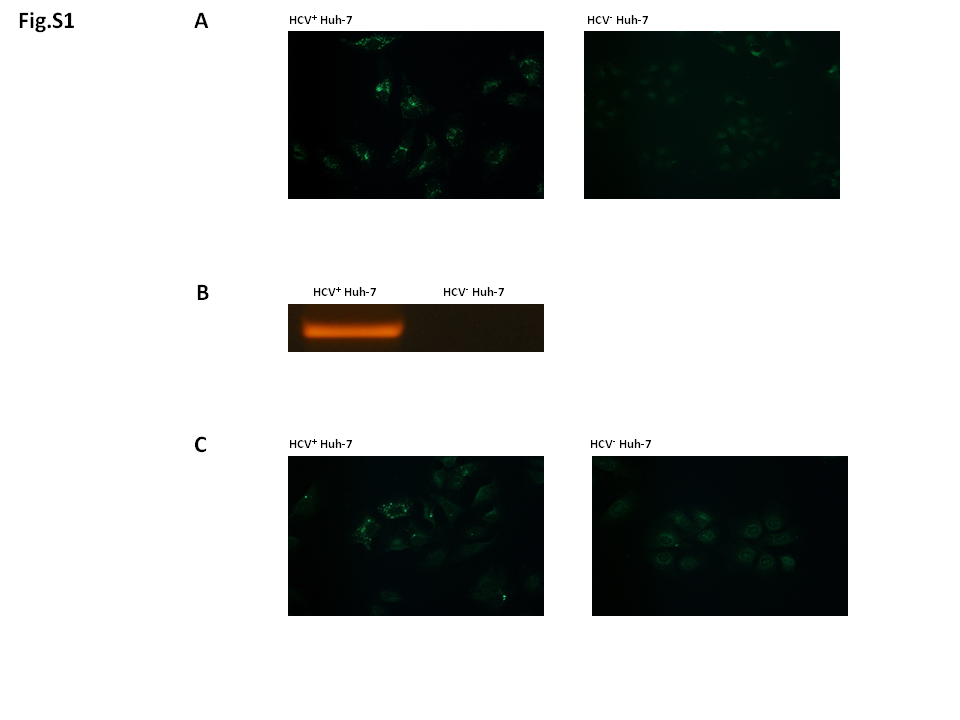

Supplement: Figure S1 — Conformation of HCV replication in hepatocyte culture system. A) Immunofluorescience staining of HCV NS5 protein in HCV-transfected Huh-7 versus mock-transfected Huh-7 cells at 48 h. B) RT-PCR of HCV core mRNA in the supernantant of HCV-transfected Huh-7 versus mock-transfected Huh-7 cells at 36 h. C) Immunofluorescience staining of HCV NS5 protein in Huh-7 cells infected by the supernatant of HCV-transfected Huh-7 cells at 48 h. (TIF) [file pone.0019664.s001.tif]

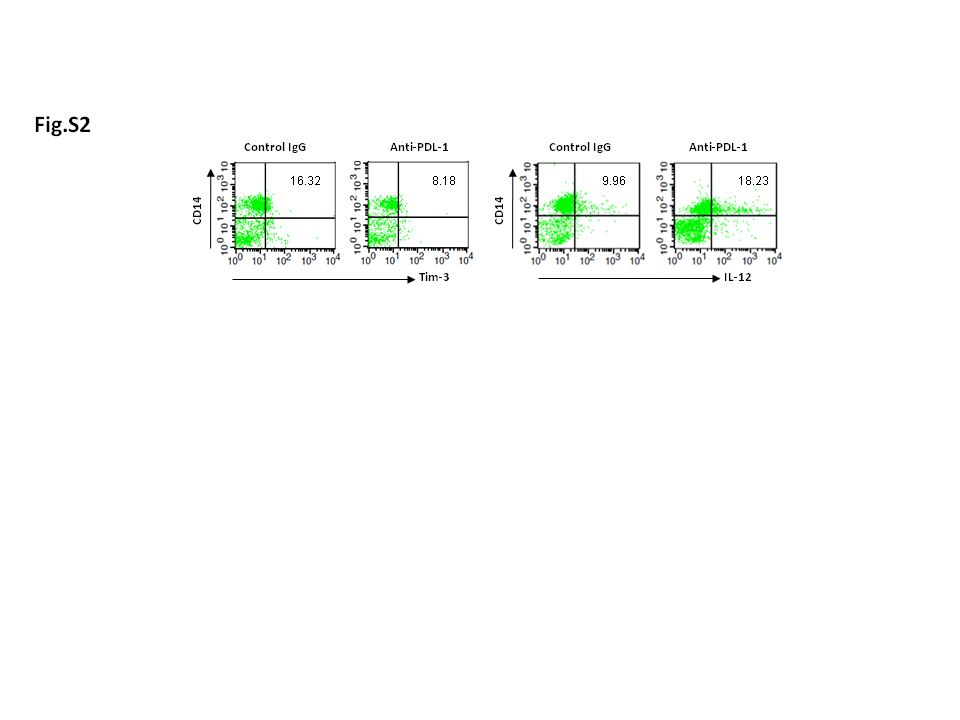

Supplement: Figure S2 — Blockade of PD-1 signaling by anti-PDL-1 antibody decreases Tim-3 expression and improves IL-12 production by CD14+ M/MØ. (TIF) [file pone.0019664.s002.tif]

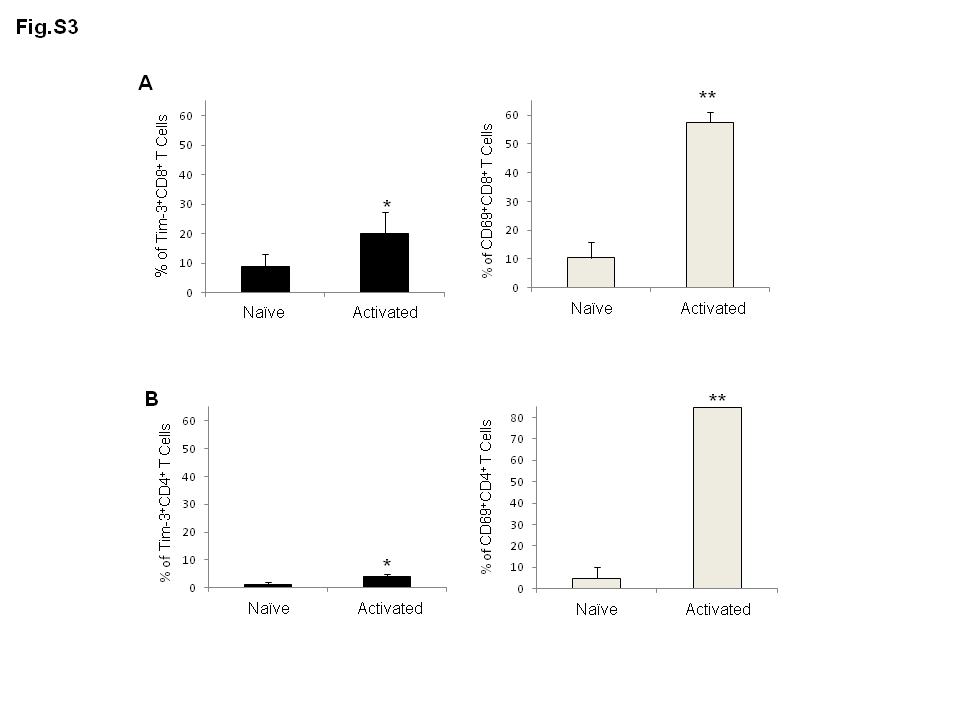

Supplement: Figure S3 — Tim-3 and CD69 expressions on naïve and activated CD4+ and CD8+ T lymphocytes. PBMC from 3 healthy subjects were stimulated with or without anti-CD3/CD28 for 24 h followed by flow cytometric analysis of Tim-3 and CD69 expressions on CD4+ and CD8+ T cells. Summary data of percentages of Tim-3+ or CD69+ cells in naïve versus activated CD8+ (A) or CD4+ (B) lymphocytes are shown, and the p value (**<0.01; ***<0.001) is denoted above the group of study subjects. (TIF) [file pone.0019664.s003.tif]
